# Surgical ergonomics - Analysis of technical skills, simulation models and assessment methods

**DOI:** 10.1186/1749-8090-10-S1-A201

**Published:** 2015-12-16

**Authors:** Sotiris C Papaspyros, Ashok Kar, David J O'Regan

**Affiliations:** 1 Cardiothoracic Surgery Department, Royal Infirmary Edinburgh, Edinburgh, EH16 4SA, UK; 2 Cardiothoracic Surgery Department, Leeds General Infirmary, Leeds, LS1 3EX, UK

## Background/Introduction

Over the past two centuries the surgical profession has undergone a profound evolution in terms of efficiency and outcomes. Societal concerns in relation to quality assurance, patient safety and cost reduction have highlighted the issue of training expert surgeons. The core elements of a training model build on the basic foundations of gross and fine motor skills.

## Aims/Objectives

We provide an analysis of the ergonomic principles involved and propose relevant training techniques. We have endeavoured to provide both the trainer and trainee perspectives.

## Method

This analysis is structured into four sections: 1) Pre-operative preparation issues, 2) technical skills and instrument handling, 3) low fidelity simulation models and 4) discussion of current concepts in crew resource management, deliberate practice and assessment.

## Results

Rehearsal, warm-up and motivation-enhancing techniques aid concentration and focus. Appropriate posture, comprehension of ergonomic principles in relation to surgical instruments and utilisation of the non-dominant hand are essential skills to master. Low fidelity models can be used to achieve significant progress through the early stages of the learning curve. Deliberate practice and innate ability are complementary to each other and may be considered useful adjuncts to surgical skills development.

## Discussion/Conclusion

Safe medical care requires that complex patient interventions be performed by highly skilled operators supported by reliable teams. Surgical ergonomics lie at the heart of any training model that aims to produce professionals able to function as leaders of a patient safety oriented culture.

